# Viral and host factors drive a type 1 Epstein–Barr virus spontaneous lytic phenotype

**DOI:** 10.1128/mbio.02204-23

**Published:** 2023-11-16

**Authors:** Katherine A. Willard, Ashley P. Barry, Cliff I. Oduor, John Michael Ong'echa, Jeffrey A. Bailey, Ann M. Moormann, Micah A. Luftig

**Affiliations:** 1 Department of Molecular Genetics and Microbiology, Duke Center for Virology, Duke University School of Medicine, Durham, North Carolina, USA; 2 Department of Pathology and Laboratory Medicine, Brown University, Providence, Rhode Island, USA; 3 Center for Global Health Research, Kenya Medical Research Institute, Kisumu, Kenya; 4 Division of Infectious Diseases and Immunology, Department of Medicine, University of Massachusetts Chan Medical School, Worcester, Massachusetts, USA; Columbia University Medical Center, New York, New York, USA; Harvard Medical School, Brigham and Women's Hospital, Boston, Massachusetts, USA

**Keywords:** Epstein–Barr virus, herpesvirus, lytic replication, burkitt lymphoma

## Abstract

Epstein–Barr virus (EBV) is a cancer and autoimmune disease-associated gamma-herpesvirus that infects the majority of adults worldwide. After initial infection, EBV generally maintains a latent state in the host with minimal viral gene expression. However, aberrations in this prototypical latent viral state leading to increased lytic replication can cause upregulation of oncogenic factors, promoting EBV-associated diseases. For example, nasopharyngeal carcinoma and post-transplant lymphoproliferative disease have strong associations with EBV lytic replication. Another EBV-associated disease, endemic Burkitt lymphoma (BL), is an extremely fast-growing cancer found primarily in children in malaria-endemic regions of Sub-Saharan Africa. Nearly all endemic BL tumors are infected with EBV and the hallmark of the disease is high levels of oncogenic c-Myc expression due to an immunoglobulin/*Myc* translocation. In this study, we describe spontaneous lytic phenotypes in EBV strains isolated from Kenyan BL patient tumors. We specifically focus on a novel Type 1 spontaneously lytic EBV strain and describe viral and host factors that could contribute to this phenotype. We found that cells infected with spontaneous lytic strains have lower c-Myc protein stability and express high levels of the plasma cell marker SLAMF7. Additionally, spontaneous lytic strains have altered latency states, which appear to further promote their lytic phenotypes. The isolation of spontaneously lytic EBV strains from endemic BL tumors provides more evidence that EBV may not maintain a prototypically latent state in patients with EBV-associated diseases and raises the possibility that promiscuous lytic activity may contribute to the formation of these diseases.

**IMPORTANCE:**

Epstein–Barr virus (EBV) infects over 95% of adults worldwide. Given its connection to various cancers and autoimmune disorders, it is important to understand the mechanisms by which infection with EBV can lead to these diseases. In this study, we describe an unusual spontaneous lytic phenotype in EBV strains isolated from Kenyan endemic Burkitt lymphoma patients. Because lytic replication of EBV has been linked to the pathogenesis of various diseases, these data could illuminate viral and host factors involved in this process.

## INTRODUCTION

The gamma-herpesvirus Epstein–Barr virus (EBV) is typically thought to maintain a tight latency in infected cells until acted upon by a reactivating stimulus, such as B-cell receptor (BCR) crosslinking or histone deacetylase inhibition (1). However, it has become more apparent through recent work that EBV can spontaneously enter the lytic cycle and that this promiscuous lytic activity may affect the risk and pathology of EBV-associated diseases (2–5). For example, increased EBV lytic replication is associated with NK/T cell lymphomas, diffuse large B-cell lymphomas, nasopharyngeal carcinoma, post-transplant lymphoproliferative disease, and Hodgkin’s lymphomas. This association may occur because cells undergoing lytic replication secrete pro-growth and inflammatory factors, such as tumor necrosis factor-α, CCL5, and IL-10, that can induce cellular transformation and oncogenesis (6). Some EBV lytic proteins also disrupt immune surveillance and antigen presentation, which can restrict the body’s response to a cell with oncogenic potential (7). In addition to these various cancers, EBV infection, and particularly lytic replication, has recently been linked to autoimmune diseases such as multiple sclerosis (8, 9).

Because of this link between EBV lytic replication and oncogenesis/autoimmune diseases, numerous recent studies have attempted to identify host cell factors that can lead to increased, spontaneous lytic EBV replication. EBV is thought to mimic the B cell germinal center reaction during infection and the establishment of latency (10, 11). One result of the germinal center reaction is the formation of plasma cells. There is evidence that lytic replication is favored in this differentiated, plasma cell-like state where cellular factors such as BLIMP1 and XBP1-s are upregulated and genes anti-correlated with differentiation, such as *EBF1*, *PAX5*, and *Bcl-6*, are downregulated (2, 12–16). Additional studies showed that STAT3 (17, 18), the nonsense-mediated decay factor UPF1 (19), and the proto-oncogene c-Myc (20) can limit lytic reactivation.

Less is understood about the viral factors that drive increased lytic phenotypes in EBV. Like all herpesviruses, EBV lytic replication occurs through a cascade of viral gene expression that begins with the immediate early (IE) lytic transcription factors *BZLF1* (ZTA) and *BRLF1* (RTA). These lytic transcription factors then induce the expression of early (E) and late (L) lytic genes that ultimately result in DNA replication and the formation of new viral particles, respectively (1, 21). Thus, the most obvious candidates for viral drivers of an increased lytic phenotype are the IE genes *BZLF1* and *BRLF1*. Indeed, it was shown that a polymorphism in the *BZLF1* promoter of all Type 2 (T2) and some rare Type 1 (T1) EBV strains increases lytic activity in these strains. This polymorphism, called Zp-V3 (22), consists of three SNPs in the Z promoter, one of which creates an NFAT binding site and, therefore, leads to increased *BZLF1* expression when bound by NFAT (3, 23). Beyond Zp-V3, variants in the EBER viral noncoding RNAs have also been linked to increased lytic activity (24).

In this study, we characterize five new EBV strains derived from Kenyan endemic Burkitt lymphoma (BL) patients. One of these new strains is a T1 strain that displays a spontaneous lytic phenotype which cannot be explained by the previously identified pro-lytic EBV polymorphisms. We therefore extensively characterized this T1 spontaneous lytic phenotype and identified viral and host factors that are involved in its resolution.

## MATERIALS AND METHODS

### Cell lines and culture

New endemic BL cell lines were isolated at the Kenya Medical Research Institute (KEMRI-Kisumu, Kenya) from fine needle tumor biopsies of Kenyan BL patients. Details of isolation and cell line establishment can be found in Saikumar Lakshmi et al. (25). Suspension cells were maintained in RPMI medium with 10–15% fetal bovine serum (FBS). After sorting, cells were maintained in media supplemented with penicillin and streptomycin. The lytic cycle was induced in P3HR1-ZHT (P3-ZHT) cells by treatment with 50 nM 4-hydroxytamoxifen (4-HT) (Sigma H7904). Cell lines used in this study have been de-identified for sharing with Duke University under a reliance agreement with the University of Massachusetts Chan Medical School, Institutional Review Board.

### Flow cytometry

To evaluate gp350 expression, cells were washed in FACS buffer [phosphate-buffered saline (PBS) with 5% FBS], then either stained with 0.25 µg of fluorophore-conjugated 72A1 antibody per 100,000 cells for 20 min at room temperature or stained with 1 µg 72A1 per 500,000 cells for 20 min at room temperature, followed by a wash in FACS buffer, then stained with 1 µg highly cross-adsorbed anti-mouse IgG Alexa-647 (Invitrogen A21235) for 15 min at room temperature. To evaluate apoptosis, cells were washed in Annexin buffer, then stained with 2 µL of Annexin V-FITC (BioLegend) and 72A1-Alexa647 as described above per 100,000 cells.

For intracellular staining (per 200,000 cells), cells were stained with Zombie Violet live-dead stain (BioLegend), fixed and permeabilized with 100 µL BD cytofix/cytoperm buffer for 10 min at 4°C, washed in BD perm/wash buffer, stained with 2 µL Ea-D antibody (Santa Cruz) for 30 min at RT, washed, then stained with 0.25 µL highly cross-adsorbed anti-mouse IgG Alexa-647 secondary antibody for 15 min at room temperature.

For absolute cell counts, equivalent volumes of samples were stained, then 10 µL of AccuCount Blank 1e6/mL 5.0–5.9 μm counting beads were added to the stained samples. During FACS data acquisition, 1,000 beads were acquired, and the number of cells was normalized to the bead number for each sample. All flow cytometry data were acquired on BD Canto machines and analyzed with FlowJo software (BD). Primary antibody information can be found in Table 1.

**TABLE 1:** Antibodies

Protein	Clone	Reactivity	Assay	Supplier	Catalog
gp350	72A1	EBV	FACS	Hybridoma	N/A
ZTA	BZ1	EBV	WB	Santa Cruz	sc-53904
RTA	N/A	EBV	WB	Argene	Discontinued
EBNA3A	N/A	EBV	WB	Exalpha	F115P
EBNA2	PE2	EBV	WB	Hybridoma	N/A
EBNA1	1EB12	EBV	WB	Santa Cruz	sc-81581
LMP1	S12	EBV	WB	Hybridoma	N/A
EBNALP	JF186	EBV	WB	Hybridoma	N/A
Magoh	21B12	Human	WB	Santa Cruz	sc-56724
Annexin V-FITC	N/A	Human	FACS	BioLegend	640906
LMP2A	14B7	EBV	WB	Santa Cruz	sc-101314
c-Myc	9E10	Human	WB	Santa Cruz	sc-40
SLAMF7/CD319-FITC	162.1	Human	FACS	BioLegend	331817
Ea-D	O261	EBV	FACS	Santa Cruz	sc-58121
gB/gp110	5B2	EBV	FACS	Santa Cruz	sc-56980

### Quantifying virus in cell supernatants

Cells were counted, washed in PBS to remove residual virus, and then seeded at equivalent densities in individual wells per time point. Upon harvest, the cells were counted, 100,000 were used in FACS to evaluate gp350 expression, then 500 µL of cell supernatant was treated with 20 µL of RQ1 DNase I (Promega) at 37°C overnight to degrade any non-encapsidated viral DNA. After digestion, the DNase I was inactivated by the addition of 30 µL proteinase K (Omega BioTek) and 100 µL of 10% SDS, followed by incubation at 65°C for 2 h. After DNase digestion, the supernatants were extracted using the Roche Viral Nucleic Acid Extraction Kit without the addition of poly A carrier. Equivalent volumes of extracted viral DNA were input into DNA qPCR reactions targeting the viral BALF5 gene (Table 2) along with a standard curve of known copy numbers of the 2089 BAC containing the EBV genome (a gift from Wolfgang Hammerschmidt, Helmholtz Munich). The qPCR data were analyzed to determine the number of viral particles produced per cell in the well at time of harvest.

**TABLE 2:** Primers

Target	Forward	Reverse	Assay	Probe
BALF5	AGCCTGCTCCTGAGAATGCT	CCACTGCTGCTGCTGTTTGA	SYBR	N/A
BALF5	CCACATGCCCTTTCCATCCT	CCTGCGTCTCATTCCCAAGT	ddPCR	N/A
Cp transcripts	AATCATCTAAACCGACTGAAGAAACAG	GAGGGGACCCTCTGGCC	TaqMan	ACCGAAGTGAAGGCCCTGGACCAAC
Wp transcripts	CGCCAGGAGTCCACACAAAT	GAGGGGACCCTCTGGCC	TaqMan	ACCGAAGTGAAGGCCCTGGACCAAC
LMP1	AATTTGCACGGACAGGCATT	AAGGCCAAAAGCTGCCAGAT	TaqMan	TCCAGATACCTAAGACAAGTAAGCACCCGAAGAT
BZLF1p-Methyl-qPCR	GATCAGGCCCTTCCATCCAC	GTTAGGGATCCGACCGGTTC	SYBR	N/A
BALF5p-Methyl-qPCR	GGATCGTGATAGCGTCTTCTG	TAGCGCTGCATGAGCAAA	SYBR	N/A
LF2p-Methyl-qPCR	ACGCTAGTGCTGCATGG	TAACGAGCGGAGAGTTGTATTG	SYBR	N/A
β-Globin-Methyl-qPCR	TAGCAACCTCAAACAGACACCA	TCACCACCAACTTCATCCAC	SYBR	N/A

### Electron microscopy

BL cells were pelleted at 300 × *g* and 2.5% glutaraldehyde in 1.0 M cacodylate buffer, pH 7.2, was added to the pellets without disturbing them. They were delivered to the Duke Electron Microscopy Laboratory for encasement in 1% molten, but cooled, 1% agar, followed by washing 30–60 min in cacodylate buffer, staining in 1% OsO_4_ in cacodylate buffer, dehydration in a graded series of acetone, and infiltration with EMBed 812 epoxy resin (Electron Microscopy Sciences, Hatfield, PA USA). After baking at 60°C for 24–48 h, they were sectioned on an ultramicrotome at 60–70 nm with a diamond knife, poststained with 2% aqueous uranyl acetate, washed in boiled distilled water followed by staining with Sato’s lead citrate and washed in water. Digital micrographs were taken on a Philips CM 12 (FEI Co., Hillsboro, OR, USA) or a JEOL2100Plus (JEOL USA, Peabody, MA, USA) electron microscope.

### Western blotting

Cells were lysed on ice in RIPA buffer (Cell Signaling Technology) supplemented with phosphatase and protease inhibitors (Roche PhosSTOP and cOmplete Tablets, respectively), sodium molybdate, and DTT. The lysates were quantified by Bradford Assay, then equal protein concentrations were denatured in Nupage buffer with 2.5% beta-mercaptoethanol at 95°C or at 72°C when probing for LMP2A. Denatured lysates were run on Novex Nupage 4–12% gradient gels (Thermo) in MOPS buffer then either transferred to PVDF membranes using the Bio-Rad Turbo Transfer system or wet transferred to PVDF at 60 V for 1 h at 4°C. Transfer efficiency was assessed with Revert total protein stain (Licor) before blocking.

Membranes were blocked in 5% milk in TBST, washed in TBST, then incubated in primary antibodies at 4°C, rocking, overnight. Primary antibody information can be found in Table 1. Primary antibodies were diluted into 5% BSA in TBST supplemented with sodium azide. Secondary antibodies conjugated to HRP were used at 1:5,000 and diluted into 5% milk in TBST. Membranes were developed using Prometheus ProSignal Femto reagent and imaged using the Licor system. When necessary, the membranes were stripped with Restore Plus western blot stripping buffer (Thermo) for 10 min then re-blocked and re-probed as above. Blots were analyzed using the Image Studio Lite software (Licor).

### Quantitative and digital droplet PCR

To evaluate differences in gene expression, RNA was isolated using either the Qiagen RNeasy kit or the Promega SV 96 total RNA isolation system and quantified by Nanodrop. The extracted RNA was then input into a cDNA synthesis reaction using the High Capacity cDNA Reverse Transcription Kit (Applied Biosystems). This cDNA was used as input for the qPCR reactions using either SYBR (2× master mix with ROX, qPCRBIO) or TaqMan (Fast Advanced Master Mix, Applied Biosystems) reagents, depending on the target (Table 2). Beta-actin expression was used for normalization in TaqMan reactions using the ACTB(DQ) 20× Oligo Mix (Applied Biosystems 4332645).

For digital droplet PCR (ddPCR) quantification of viral stocks, DNA was extracted from 200 µL virus preparations using the Roche Viral Nucleic Acid Kit. The extracted DNA was diluted 1:10 and 1:100, then used as the template for ddPCR with 2× QX200 EvaGreen ddPCR Supermix (Biorad) targeting the EBV BALF5 gene on a Bio-Rad QX200 Droplet Digital PCR system.

### Methylation-specific qPCR

To assess methylation differences at CpG sites in EBV promoters, we used the methyl-qPCR assay detailed in Borde et al. (26). Briefly, gp350 FACS was performed as previously described, then 2 million cells were collected and DNA was extracted using the Invitrogen PureLink Genomic DNA mini kit. The resulting DNA (containing both host and viral DNA) was digested with 100U of either HpaII or MspI per microgram of DNA at 37°C overnight. HpaII only cleaves CCGG sites if the site is methylated, whereas its isoschizomer MspI cleaves both methylated and unmethylated CCGG sites. We then performed qPCR on the digested samples compared to undigested samples using primers specific to CCGG sites in the *BZLF1*, *BALF5*, and *LF2* promoters. We used primers to β-Globin for normalization and calculated the methylation index as described in the original methods paper (26). The primer sequences can be found in Table 2.

### Virus stock generation and EBV infections

To produce viral stocks from the BL cell lines, cells were treated with 24 ng/mL TPA and 4 mM sodium butyrate for 3 days then pelleted. The cell supernatant was passed through a 0.45-µm filter then centrifuged at 4°C/10,000 RPM for 3 h in a Beckman J2-HS centrifuge to pellet the virus. After centrifugation, the supernatant was removed, and the virus pellet was resuspended in RPMI + 10% FBS at 1/100 of the original culture volume to make a 100× stock.

For infections, peripheral blood mononuclear cells (PBMCs) were harvested via Ficoll gradient from whole blood obtained from Gulf Coast Regional Blood Center. For proliferation tracking experiments, the PBMCs were stained with CellTrace Violet (CTV, Thermo) for 20 min at 37°C, then rested overnight. All infections occurred the day after PBMC isolation. For bulk infections, 10 million PBMCs were infected with 1 mL of EBV stocks for 1 h at 37°C with occasional rocking. After 1 h, the virus supernatant was removed and the cells were resuspended in RPMI + 15% FBS + cyclosporine A at 1 million PBMCs/mL. For transformation assays, the PBMCs were plated at 100,000 cells/well in 96-well plates, then infected with serial dilutions of virus stocks (between 15 and 0.0015 µL of virus per well).

During transformation, the cells were incubated at 37°C and fresh media was added periodically as needed. Transformation was complete by 5 weeks after infection. At this point, bulk LCLs were maintained in culture and transformation assay plates were scored for outgrowth (i.e., the presence of clumped LCLs in each well). After scoring the transformation assays, LCL clones were picked from the wells infected with the lowest volume of virus where outgrowth occurred and scaled up to produce clonal LCLs.

We used RT-qPCR for Wp transcripts 3 days after infection to normalize the infectivity of the different viral stocks. PBMCs from two different blood donors were infected with serial dilutions of the viral stocks. After 3 days, cDNA was made from the infected cell RNA and used in qPCR reactions for Wp transcripts normalized to actin (see Table 2 for primer info). The Wp expression for the different strains was normalized to that of B95-8.

### Flow cytometry-based cell sorting

To sort based on gp350 expression, cells were stained with 72A1 conjugated to Alexafluor-647 then sorted into pure gp350(+) and gp350(−) subpopulations by the Duke Cancer Institute Flow Cytometry Core. The sorted cells were returned to culture and monitored for gp350 expression after sorting. Cells were evaluated for original gp350 expression by the residual 72A1-A647 signal and new gp350 expression by staining with 72A1 conjugated to Alexafluor-488, followed by FACS analysis. Total gp350(+) cell percentage daily after sorting was determined by the addition of the 72A1-A647 and 72A1-A488 signals.

### Live-cell microscopy

Cells sorted into gp350(+) and gp350(−) subpopulations were seeded into 35 mm glass bottom dishes (Mattek) with a 250-µm silicone microcell array (Microsurfaces) attached to the bottom of the dish. The microcell array was sterilized with 70% ethanol before seeding 20,000 cells in 2 mL media directly on top of the array. The cells were allowed to settle into the wells of the microcell array before the dishes were loaded into an Olympus LCV110U VivaView Live Cell Microscope incubator. Ten fields of view each encompassing a single well on the microarray were chosen per sample and images were acquired every 30 min for 96 h.

### Proliferation tracking in BL cells

One million BL cells were resuspended in 1 mL PBS and 1 µL of 0.5 mM CTV was added to the suspension. The cells were incubated at 37°C for 20 min with periodic mixing. The cells were washed in PBS with 5% FBS to quench the staining reaction. The cells were resuspended in RPMI + 15% FBS and rested for approximately 30 min. The cells were then stained for gp350 using 2.5 µg of 72A1-A647 and returned to culture. CTV and gp350 expression were checked by FACS immediately after staining and then monitored daily for 4 days thereafter. On days 1–4 following staining, approximately 100,000 cells were removed from the culture and stained with 72A1-A488 to differentiate between original and newly gp350(+) cells.

### EBV genomic sequencing library preparation

DNA was isolated from the BL cell lines using the Monarch DNA purification kit (NEB). Sequencing libraries were then directly prepared from the DNA isolated from the cell lines, as previously described by Kaymaz et al. (27). Briefly, the Illumina sequencing library preparation steps consisted of DNA shearing, blunt-end repair (Quick Blunting kit; NEB), 3′-adenylation (Klenow fragment 3′ to 5′ exo-; NEB), and ligation of indexed sequencing adaptors (Quick Ligation kit; NEB). We PCR amplified the libraries to a final concentration with 10 cycles using KAPA HiFi HotStart ReadyMix and quantified them using the qubit and fragment analyzer. We then pooled the sample libraries from each cell line, balancing them according to their EBV content, measured by duplex EBV viral load qPCR (28), and proceeded to target enrichment hybridization using custom EBV-specific biotinylated RNA probes (MyBaits; Arbor Biosciences) (29), to enrich for EBV specific libraries. The libraries were then sequenced on the Illumina NextSeq 550 platform, as paired-end 150 bp libraries (Illumina, Inc.).

### EBV sequence preprocessing and *de novo* genome assembly

We checked the sequence quality using FastQC (v0.10.1) (30), then trimmed residual adapter sequences and low-quality bases (<20) using cutadapt (v1.7.1) (31) and prinseq (v0.20.4) (32), respectively. After removing reads that mapped to the human genome (hg38), we *de novo* assembled the remaining reads into contigs with VelvetOptimiser (v2.2.6) (33) using a kmer search ranging from 21 to 149 to maximize N50. We then ordered and oriented the contigs guided by the reference genomes (NC_007605.1 for Type 1 and NC_009334.1 for Type 2) using ABACAS, extended with read support using IMAGE (34), and merged the overlapping contigs to form larger scaffolds. By aligning reads back to scaffolds, we assessed contig quality requiring support from ≥10 unique reads. We created a final genome by demarcating repetitive and missing regions due to low coverage with sequential ambiguous “N” nucleotides.

### Plasmids and overexpression experiments

pcDNA3 encoding B95-8 *BRLF1* (pcDNA3-B958-RTA) was a gift from Eric Johannsen (University of Wisconsin-Madison). We used site-directed mutagenesis to construct pcDNA3-RTA containing the four coding mutations present in strain 720 (pcDNA3-720-RTA). Plasmids encoding the LMP2A coding sequences from strains B95-8 and 720 under control of a CMV promoter were synthesized by Twist Biosciences. All plasmid inserts were verified by Sanger sequencing before use in experiments.

We used the Neon electroporator (Thermo) to overexpress RTA and LMP2A in P3HR1-ZHT and Mutu-I cells. Two million cells per condition were resuspended in Belzer solution then mixed with the relevant plasmids. pcDNA3.1 encoding mGreenLantern was included in each reaction as a marker of transfection efficiency. For each sample, we electroporated two times using 100 µL Neon tips with the following settings: 1350 V, 40 ms, and 1 shock. The transfected cells were plated in RPMI + 15% FBS. The next day, approximately 200,000 cells were used for FACS analysis of lytic induction by detection of transfected cells (GFP+) expressing the early lytic antigen Ea-D. The remaining cells were collected to confirm RTA and LMP2A expression by western blot.

### RNA sequencing

Total RNA was isolated from BL cell lines using the Qiagen RNeasy kit with DNase digestion. Novogene prepared the RNA sequencing libraries, performed the sequencing on an Illumina Novaseq with 150 bp paired-end reads, and did the initial bioinformatic analysis. We supplied Novogene with a reference genome consisting of hg38 concatenated with an annotated Type 1 EBV genome (NC_007605), which allowed for the alignment of both human and EBV reads. HISAT2 (35) was used for alignment and DESeq2 (36) was used for differential gene expression (DEG) analysis. The resulting DEG data were further analyzed by our lab.

### Genetic and sequencing analyses

EBV genetic alignments and analyses were performed with the Geneious Prime software. Phylogenetic analyses were performed with Geneious using the MAFFT aligner with FFT-NS-1 algorithm and trees were constructed with Geneious Tree Builder and the Tamura-Nei neighbor-joining model with B95-8 as the outgroup. RNA sequencing analyses and data visualization were performed using R (version 4.2.1) and ggplot packages.

## RESULTS

### New endemic BL-derived EBV strains are spontaneously lytic

**Fig 1:**
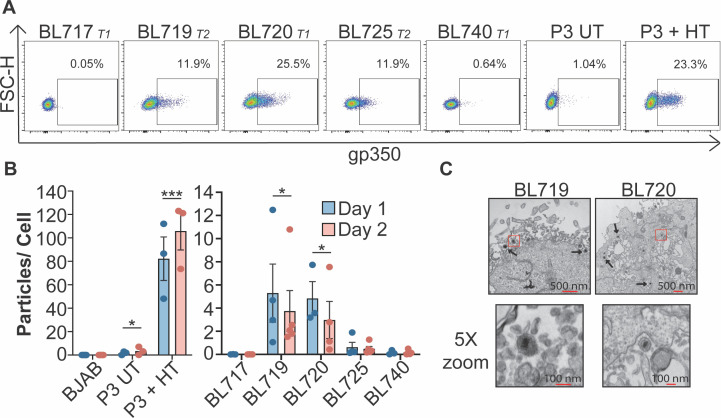
New EBV strains are spontaneously lytic. (**A**) Gp350 FACS of newly established EBV-infected BL cell lines (BL717, BL719, BL720, BL725, and BL740) compared to untreated and P3-ZHT cells treated with 50 nM 4-HT. The EBV strain genetic type and the percentage of gp350(+) cells in the lymphocyte, single-cell population are noted. (**B**) EBV particles produced per cell 1 and 2 days after plating. DNase-resistant viral particles in the supernatants were quantified by DNA qPCR for the viral BALF5 gene and a standard curve made from the 2089 BAC harboring the EBV genome. A two-way ANOVA test revealed that strain is significant but day is not. Subsequent unpaired *t* tests show significant increase in particle production from UT and 4HT-treated P3-ZHT, BL719, and BL720 compared to BJAB but no significant difference from BL717, BL725, and BL740 compared to BJAB; *P < 0.05, ***P < 0.009. (**C**) Electron micrographs of EBV particles in BL719 and BL720. The boxed portion of the top images is shown at 5× zoom in the bottom images.

Establishment and characterization of five new BL cell lines derived from unique patients has been described previously (25). Each BL cell line is also infected with different strains of EBV that include both Type 1 (T1) and Type 2 (T2) genetic variants of EBV (37). For consistency, we have named these new viral strains with the same de-identification number as the BL tumors from which they are derived (i.e., strain 719 is derived from BL719). Interestingly, the EBV strains in three out of five of these new BL cell lines spontaneously enter the lytic cycle at a high level as measured by FACS for the major viral glycoprotein, gp350, which is displayed on the surface of a lytic cell (Fig. 1A). More than 11% of the BL cells infected with strains 719, 720, and 725 are gp350(+). This level of gp350 expression is similar to that in P3HR1-ZHT (P3-ZHT) cells induced into the lytic cycle by 50 nM 4-HT treatment. However, it is notable that this level of lytic activity in the BL cells occurs spontaneously within the culture.

EBV is known to spontaneously reactivate at a low level in culture, but the degree of spontaneous reactivation we observed in these new BL cells was much higher than expected (2–4, 38). Therefore, we determined if these spontaneous lytic BL cells are entering the full lytic cycle and producing viral particles rather than erroneously expressing gp350. To address this question, we measured if viral particles accumulate in the cell supernatant using an established DNA qPCR assay that detects expression of the viral gene BALF5 (20). Through this method, we determined the number of viral particles produced per cell within the BL cell culture (Fig. 1B). Indeed, the gp350(+) strains 719 and 720 accumulate DNase-resistant viral particles in the cell supernatant, while the EBV-negative BJAB cell line and the gp350(−) BL cells 717 and 740 do not. It is notable, however, that BL719 and BL720 shed about 10-fold fewer viral particles compared to the induced lytic P3HR1 cells, suggesting a lower burst size for spontaneous compared to induced lytic reactivation. Although BL725 is gp350(+), we did not detect viral particles in the cell supernatant, suggesting that this line may undergo an abortive lytic replication that does not result in the formation of intact viral particles, which has been previously described in some strains (39, 40). These data are corroborated with electron microscopy, where we detected EBV particles produced from BL719 and BL720 cell lines but not from BL725 (Fig. 1C).

### The T1 lytic phenotype diminishes over time in culture

**Fig 2:**
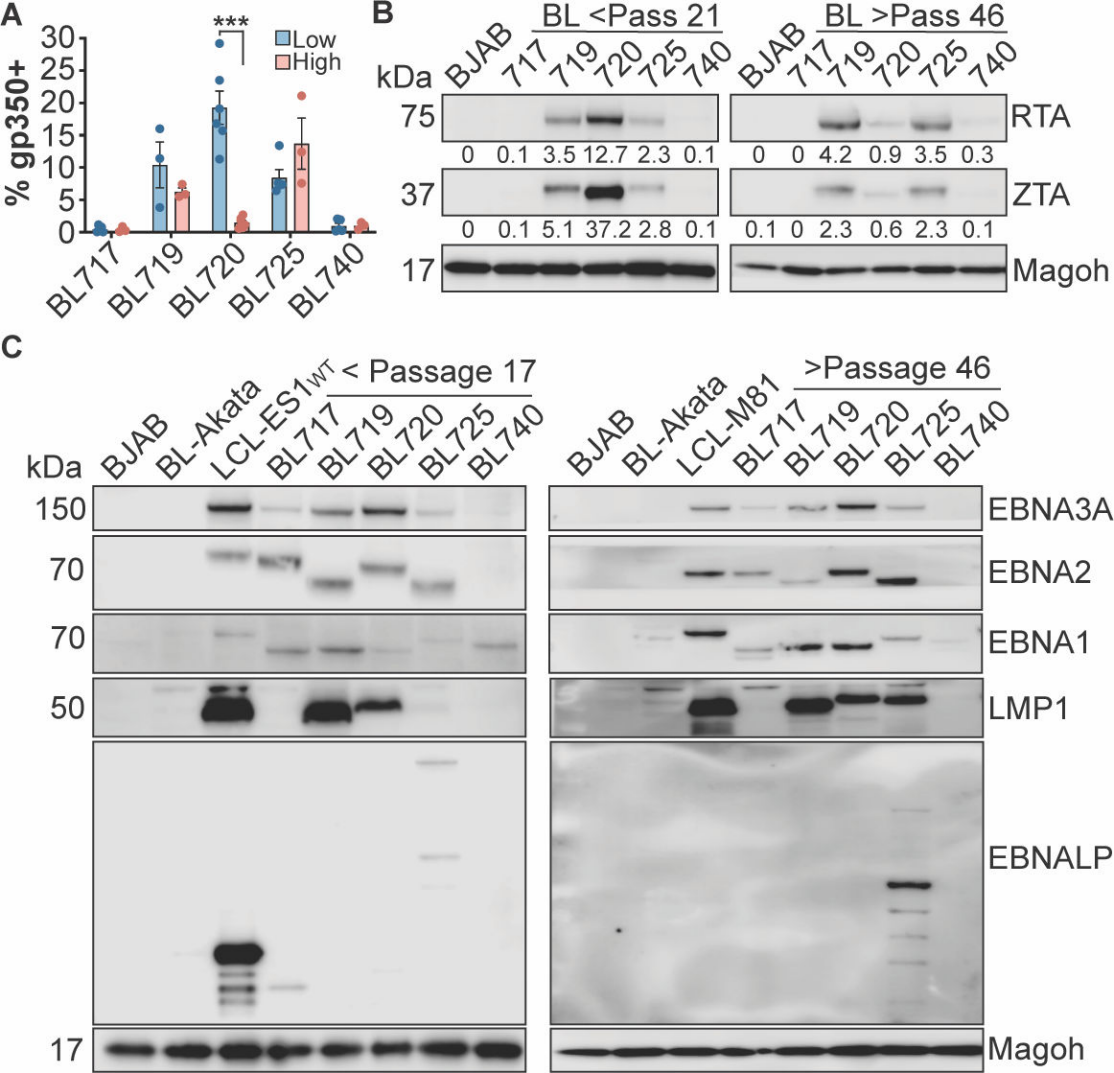
Lytic and latency states of the new EBV strains. (**A**) Gp350 FACS results from low (<passage 25) and high (>passage 58) passage BL cell lines reveal that the spontaneous lytic phenotype in BL720 is significantly diminished over time in culture. ***P < 0.0001. (**B**) Western blots of the immediate early lytic transcription factors RTA and ZTA from low passage (left) and high passage (right) cell lysates. The numbers under the blots indicate the percentage of viral protein expression compared to the cellular protein Magoh. BJAB-EBV-negative B cell line. (**C**) Western blot of EBV latency proteins expressed in the low passage (left) and high passage (right) BL cell lysates. BL-Akata is a latency I control, LCL-ES1-WT and LCL-M81 are latency III controls, and BJAB is an EBV-negative B cell line. Only presence vs absence of protein should be interpreted (rather than relative expression between targets) as the contrast was adjusted for each target to better visualize the bands.

Although the T1 spontaneous lytic phenotype is very strong in early passage BL720, we noticed that the phenotype diminishes over time in culture. The percentage of gp350(+) cells in BL720 drops significantly in high passage (>p58) BL720 while there is no significant difference in the proportion of lytic cells in the T2-infected BL719 and BL725 (Fig. 2A). This finding is supported by decreased expression of the immediate early viral lytic transcription factors ZTA and RTA in high passage BL720 cells but not in high passage BL719 or BL725 (Fig. 2B). We hypothesized that BL720 lytic cells may be more apoptotic compared to lytic BL719 and BL725 cells. To test this hypothesis, we analyzed the expression of gp350 and annexin V by FACS. We found that BL720 cells do not express a higher proportion of annexin V and gp350 double-positive cells compared to the T2 spontaneous lytic lines BL719 and BL725, suggesting that there is another mechanism of lytic silencing that is not tied to selective apoptosis of the lytic cells (Fig. S1A).

Epigenetic silencing through promoter methylation is a known regulatory mechanism of the EBV lytic cycle (41–44). Therefore, we employed a methylation-specific qPCR technique (26) to determine if CpG sites within the *BZLF1*, *BALF5*, and *LF2* promoters are differentially methylated. As expected, we generally found that methylation at these sites is inversely correlated with gp350 expression, with a higher methylation index present in the latent samples. The notable exception is with BL725, where although the cells are gp350(+), the lytic promoters are in a more methylated state. This observation correlates with our inability to detect viral particles shed from BL725 (Fig. 1B and C). Importantly, we observed a significant increase in methylation in the latent BL720 cells compared to the lytic BL720 cells in the *BALF5* and *LF2* promoters and increased, although not statistically significant, methylation in the *BZLF1* promoter (Fig. S1B). Thus, lytic gene promoter methylation appears to be a silencing mechanism of the T1 spontaneous lytic phenotype.

### The BL-associated EBV strains have unusual latency states

Because the new EBV strains exhibit unexpected lytic protein expression, we hypothesized that latency protein expression would also be atypical. In BL cells, EBV is typically thought to exist in the latency I state, where only EBNA1 and the EBER and BART noncoding RNAs are expressed (45). However, there are reports of unusual latency states in BL cell lines and endemic BL tumors, including Wp-restricted and latency III (46–49). Of particular note, a study of EBV gene expression in endemic BL tumors from Malawian patients revealed the expression of *LMP1* and *LMP2A* in addition to *EBNA1*. Furthermore, these tumors also expressed the lytic proteins like ZTA and LF3 (49). Surprisingly, BL740 is the only cell line in our panel that exhibits the prototypical BL latency I state (Fig. 2C). All three spontaneous lytic lines (BL719, BL720, and BL725) express high levels of LMP1 and many of the EBNA proteins. In fact, BL725 expresses EBNA-LP which is characteristic of a latency III/ LCL state. This is noncanonical but perhaps unsurprising given that the latency III program can be activated during the lytic cycle (21). BL717 expresses EBNAs 1, 2, and 3A but not the LMPs, putting it in a latency IIb-like state (45, 46, 50). The size difference in EBNA2 for BL719 and BL725 is due to their being T2 EBV strains; much of the variation between T1 and T2 EBV lies in the EBNA2/3 region (37). Interestingly, despite the lytic silencing in BL720, the latency III state of this line does not change over time in culture (Fig. 2C, right). These data indicate that these new BL-derived EBV strains not only have high spontaneous lytic activity, but also exist in unexpected, but not unprecedented, latency states.

### Spontaneous lytic phenotypes are correlated with high c-Myc protein turnover

**Fig 3:**
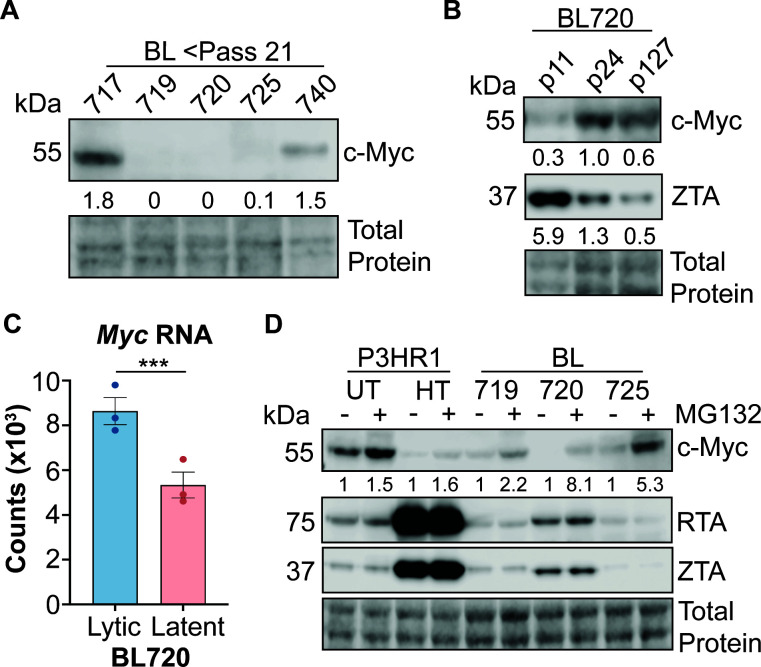
Lytic BL Cells have lower c-Myc stability. (**A**) Western blot of c-Myc protein expression in low passage, lytic BL cells. The lysates in this blot are the same as those in Fig. 2B left panel. The numbers under the blot are c-Myc expression as a percentage of total protein. (**B**) Western blot of c-Myc and ZTA protein expression in low, mid, and high passage BL720. The numbers under the blots are c-Myc expression as a percentage of total protein. (**C**) *Myc* RNA expression counts from lytic vs latent BL720 RNA sequencing experiment. *** indicates an adjusted *P* value of 0.007. (**D**) Cells plated the previous day were either treated with 10 µM of MG132 or DMSO (UT) for 2 h then harvested to assess c-Myc stability. P3-ZHT cells were treated with 50 nM 4-HT at the time of plating (~24 h 4-HT treatment before addition of MG132). The numbers under the blot are normalized c-Myc expression relative to untreated cells within each cell type (i.e., there is a 5.3× relative increase in c-Myc expression upon MG132 treatment in BL725 compared to untreated BL725). Note that there is no apparent difference in RTA or ZTA expression upon MG132 treatment as 2 h of proteasome inhibition is likely too short of a time to affect turnover of these proteins.

A recent study demonstrated that the oncoprotein c-Myc is involved in the regulation of the latent to lytic switch in EBV (20). We therefore hypothesized that a similar mechanism may occur in our BL cells and assayed c-Myc expression by western blot. There is a very strong anticorrelation between lytic and c-Myc protein expression; c-Myc is almost undetectable in the lytic BL cell lysates (Fig. 3A; same lysates as in Fig. 2B, left). We were surprised by this result because high c-Myc expression is a hallmark of Burkitt lymphoma due to the translocation of immunoglobulin promoter to the c-Myc gene (51). Additionally, these BL cell lines were screened for *IgH/L-Myc* translocations and officially diagnosed as endemic Burkitt lymphomas (25). To reconcile this dichotomy between expected and observed c-Myc abundance, we also assayed c-Myc and ZTA protein expression in low, mid, and high passage BL720 cells. We found that c-Myc protein expression increases with passage and that this is anti-correlated with ZTA expression level (Fig. 3B). Interestingly, when we checked expression counts of *Myc* RNA from RNA sequencing of lytic vs latent BL720 samples, we found that *Myc* RNA expression is significantly higher in the lytic BL720 samples despite the lower c-Myc protein expression in these samples (Fig. 3C; log2FC between lytic vs latent samples = 0.7).

We therefore hypothesized that c-Myc protein is less stable in lytic BL720 cells compared to the higher passage, latent BL720. To test this hypothesis, we treated the cells with MG132 proteasome inhibitor to assay if c-Myc is degraded more rapidly in the lytic compared to latent cells. Indeed, treatment with 10 µM of MG132 for 2 h was sufficient to stabilize c-Myc expression, demonstrating that c-Myc protein turnover is higher in cells undergoing lytic replication (Fig. 3D). Interestingly, this result is true in both the spontaneous lytic BL cells as well as in the induced lytic P3-ZHT system; treatment with 50 nM 4-HT for 24 h dramatically reduced c-Myc expression compared to untreated cells and MG132 treatment stabilized c-Myc to an extent, though not back to expression levels observed in untreated cells. These results add further support that c-Myc is broadly anti-correlated with lytic activity across both T1 and T2 EBV strains as well as in spontaneous and induced lytic systems.

### BL720 cells revert to a basal state after sorting to a pure gp350(+) population

**Fig 4:**
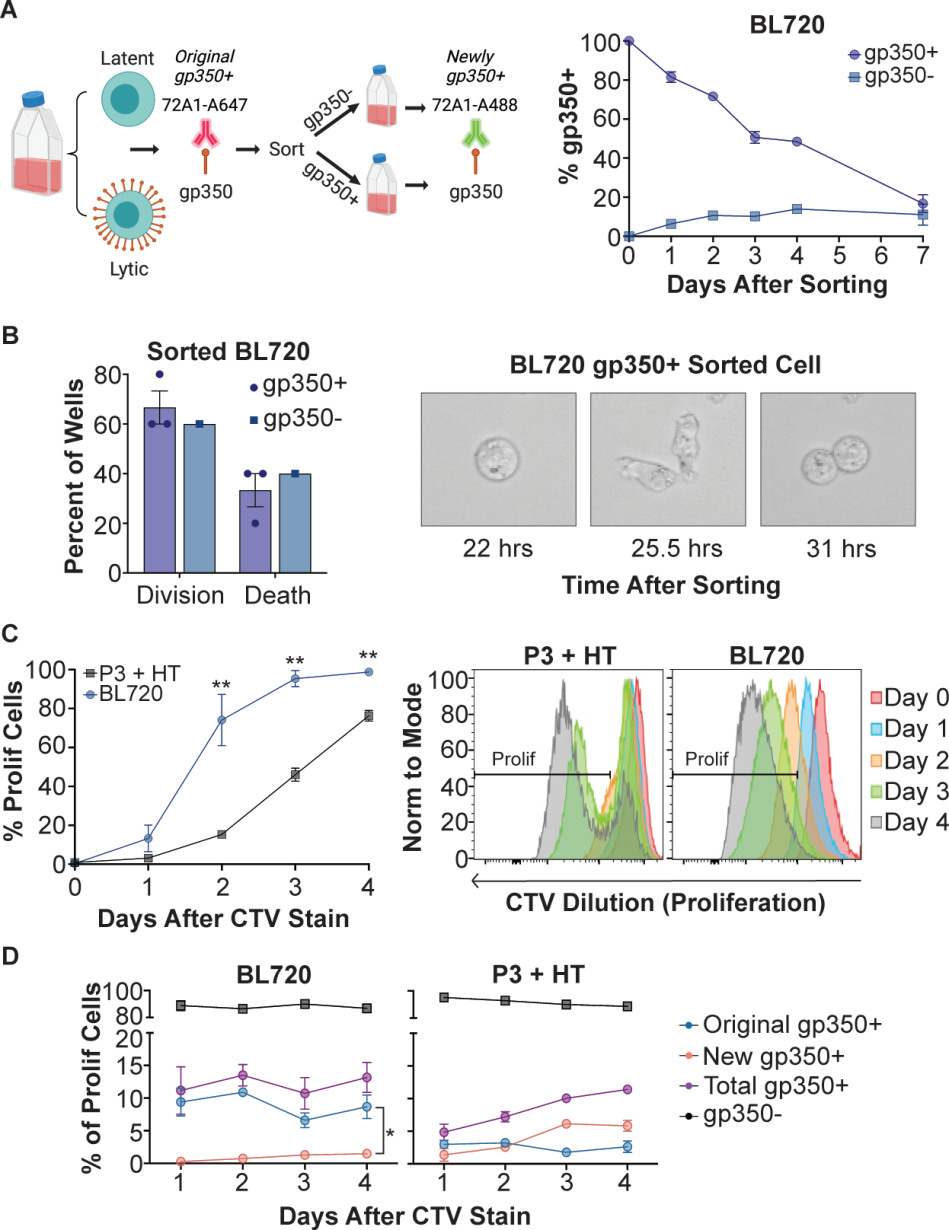
BL720 reverts to a basal lytic state after sorting into gp350±subpopulations. (A) Left—Schematic of sorting and follow-up FACS strategy (made with Biorender). Right—Percentage of total gp350(+) cells (both original and new) over time following sorting of BL720 into pure gp350(+) and gp350(−) subpopulations. (B) Left—BL720 cells from the d0 sorted populations in panel (A) were seeded into live cell imaging plates and 10 wells were recorded for each sample. The data displayed are the percentage of these 10 wells where any cell in the well divided (division) vs the percentage of the wells where the cells died without any division occurring (death). *N* = 3 for gp350(+) sorted cells, *N* = 1 for gp350(−) sorted cells. Right—Live cell image of a cell from the gp350(+) sorted population dividing. (C) Left—Proliferation of low passage (lytic) BL720 compared to P3-ZHT cells stimulated with 4-HT. Proliferation was measured by CTV dilution over time. Right—Representative proliferation FACS plots of the graphical data. (D) Proportions of lytic vs latent subpopulations in the proliferating cells in panel (C). Similar to the sorting strategy in panel (A), cells were stained with 72A1-A647 immediately after CTV staining then with 72A1-A488 on the days after CTV staining. This staining strategy allows tracking of cells that were gp350(+) then subsequently divided (original gp350+) vs cells that divided then became gp350(+) (new gp350+). Total gp350(+) is the sum of the original and new gp350(+) signals. There were significantly more proliferating original gp350(+) cells compared to newly gp350(+) cells in BL720 at day 4 after CTV staining (*P* = 0.0164). In the P3 + HT samples, there were relatively more proliferating new gp350(+) cells compared to original gp350(+), though this difference was not quite significant (*P* = 0.0578).

We next investigated the outcome of sorting BL720 cells into pure gp350(+) and gp350(−) subpopulations by FACS then returning them to culture. We observed the gp350(−) sorted population re-establish a basal level of gp350 positivity after 3–4 days, which provides more support to the spontaneous nature of reactivation in these cells (Fig. 4A). Surprisingly, instead of dying, as one might anticipate for a cell undergoing viral lytic replication, we observed the gp350(+) isolated population quickly became more gp350(−), thus re-establishing the basal level of gp350 positivity seen in the starting bulk culture. We confirmed that another viral glycoprotein, gB/gp110, is expressed at a similar level as gp350 in BL720, suggesting that our sorting approach collected genuine gp350-expressing cells and did not detect bound viral particles or secreted gp350 (Fig. S2). We also used live cell microscopy to track the subpopulations after sorting and observed that gp350(+) cells divide at equal frequencies as the gp350(−) cells (Fig. 4B; Videos ). Because we observed that spontaneously gp350(+) cells can divide, we used CTV staining to measure the proliferation of BL720 compared to 4HT-treated P3-ZHT cells. This experiment revealed that, at the population level, BL720 proliferates significantly faster than P3-ZHT cells induced with 4HT (Fig. 4C). We also stained these cells with the 72A1 antibody immediately after CTV staining to determine if gp350(+) cells proliferate. When gating specifically on proliferating cells, we observed that BL720 spontaneous gp350(+) cells can proliferate and do so more than P3-ZHT stimulated gp350(+) cells (Fig. 4D). Specifically, the gp350(+) cells that were initially present in the BL720 culture (original gp350+) proliferate resulting in a significantly higher proportion of these cells at day 4 compared to cells that proliferated then became gp350(+) (new gp350+). In contrast, there is a larger proportion of newly gp350(+) cells in the proliferating P3-ZHT cells, suggesting that a gp350(−) cell proliferated then went lytic rather than a cell that was already gp350(+) proliferating. Taken together, these data provide evidence that spontaneously lytic BL720 cells can divide and proliferate and that there is a homeostatic level of spontaneous lytic cells maintained in BL720 at low passage numbers in culture.

### The spontaneous lytic EBV strains are transforming *in vitro*

**Fig 5:**
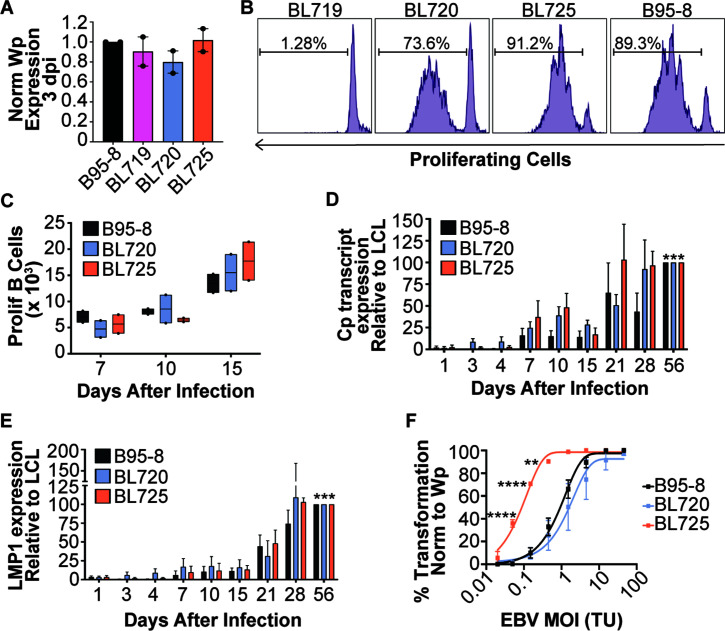
Spontaneously lytic BL-derived strains are transforming *in vitro*. (**A**) Infectivity of viral stocks derived from BL719, BL720, and BL725 compared to B95-8 as measured by qPCR for Wp transcripts 3 days after infection of PBMCs from two blood donors. The qPCR results are displayed as Wp equivalents for 100 µL of virus per 1 million PBMCs normalized to B95-8. (**B**) Representative FACS plot of proliferating B cells 7 days after infection with the indicated strains. The percentage of proliferating B cells is noted on the plots. (**C**) Number of proliferating B cells 7, 10, and 15 days after infection of PBMCs from two donors with the indicated strains. A two-way ANOVA analysis shows a significant increase in the number of proliferating B cells over time after infection (*P* = 0.0128) but no significant differences between strain or PBMC donor. (**D**) qPCR for EBV C promoter (Cp) derived transcripts infection of three PBMC donors with the indicated strains. Cp transcript abundance was measured periodically early after infection until LCLs were formed by day 56 post-infection. The data are normalized to the expression level in the LCL state at day 56. A two-way ANOVA analysis indicates significant differences in Cp expression between days after infection (*P* < 0.0001) and between PBMC donors (*P* = 0.0013), but not between strains. Subsequent paired *t* tests show a significant increase in Cp transcript expression for each strain between days 1 and 56 (B95-8 *P* = 0.0004; BL720 *P* = 0.0003; BL725 *P* = 0.0006). (**E**) LMP1 transcript expression from the same samples as in (D). A two-way ANOVA analysis indicates a significant difference in LMP1 expression between days after infection (*P* = 0.009) but not between strain or donor. Subsequent paired *t* tests show a significant increase in LMP1 transcript expression for each strain between days 1 and 56 (B95-8 *P* = 0.0003; BL720 *P* = 0.0002; BL725 *P* = 0.0005). (**F**) Transformation assay of three PBMC donors infected with the indicated strains at the indicated MOIs. Infections were done with equivalent volumes of virus then normalized to Wp equivalents as in (A). One-way ANOVAs at the discrete MOIs show that strain BL725 is significantly more transforming than strains B95-8 and BL720, while there is no significant difference in transformation between strains B95-8 and BL720.

EBV infects naïve B cells and transforms them into indefinitely proliferating lymphoblastoid cell lines (LCLs) *in vitro* (52), providing a useful tool to study EBV infection characteristics and phenotypes. Therefore, we next tested if virus produced from the spontaneous lytic BL cell lines 719, 720, and 725 could transform B cells into LCLs. We treated BL719, BL720, and BL725 with TPA and sodium butyrate to further induce the lytic cycle, which resulted in viral stocks that had similar infectivities and titers as the common laboratory strain B95-8 as assessed by Wp transcript expression 3 days after infection (Fig. 5A) and digital droplet qPCR for the EBV BALF5 gene (Fig. S3).

We then used our new viral stocks to infect PBMCs isolated from whole blood. EBV infection of B cells *in vitro* induces hyperproliferation of a subset of the cells in the first week of infection that can be measured by dilution of proliferation tracking dyes like CTV (53, 54). Strains 720 and 725 induce B cell proliferation 7 days after infection to a similar level as B95-8, but strain 719 does not induce proliferation as measured by CTV dilution (Fig. 5B). Strain 719 is a T2 strain, and these are known to be less transforming *in vitro* due to polymorphisms in the EBNA2 gene relative to T1 strains (55). However, 725 is also a T2 strain that induces B cell proliferation at an equivalent degree to the T1 strains. We also tracked the absolute number of proliferating B cells over time after infection and observed a steady increase in proliferating B cells through 15 days after infection (Fig. 5C).

We collected RNA samples periodically during early hyperproliferation through the LCL state (56 days after infection) and observed significant accumulation of transcripts originating from the C promoter (Fig. 5D) and LMP1 transcripts (Fig. 5E), indicating that infection with these strains results in the formation of LCLs. Additionally, we observed higher Cp transcript expression compared to LMP1 expression early in infection (Fig. 5D and E; 7–10 days), indicating that these new EBV strains also induce a latency IIb state that our lab has previously described with B95-8 (45, 50).

Finally, we performed a transformation assay where PBMCs were infected with equivalent limiting dilution volumes of virus and outgrowth was measured 5 weeks after infection. These results indicate that when cells are infected with an equivalent multiplicity of infection (MOI) of virus (as determined by Wp-equivalents), strain 720 transforms equally well as B95-8 and strain 725 transforms significantly more efficiently at low MOIs (between 0.1 and 1) than strains 720 or B95-8, despite it being a T2 strain (Fig. 5F). Overall, these data indicate that the new BL-derived strains 720 and 725 transform B cells into LCLs at an equivalent or higher level than the standard lab strain B95-8.

### Spontaneous lytic EBV strains transform B cells into spontaneous lytic LCLs

**Fig 6:**
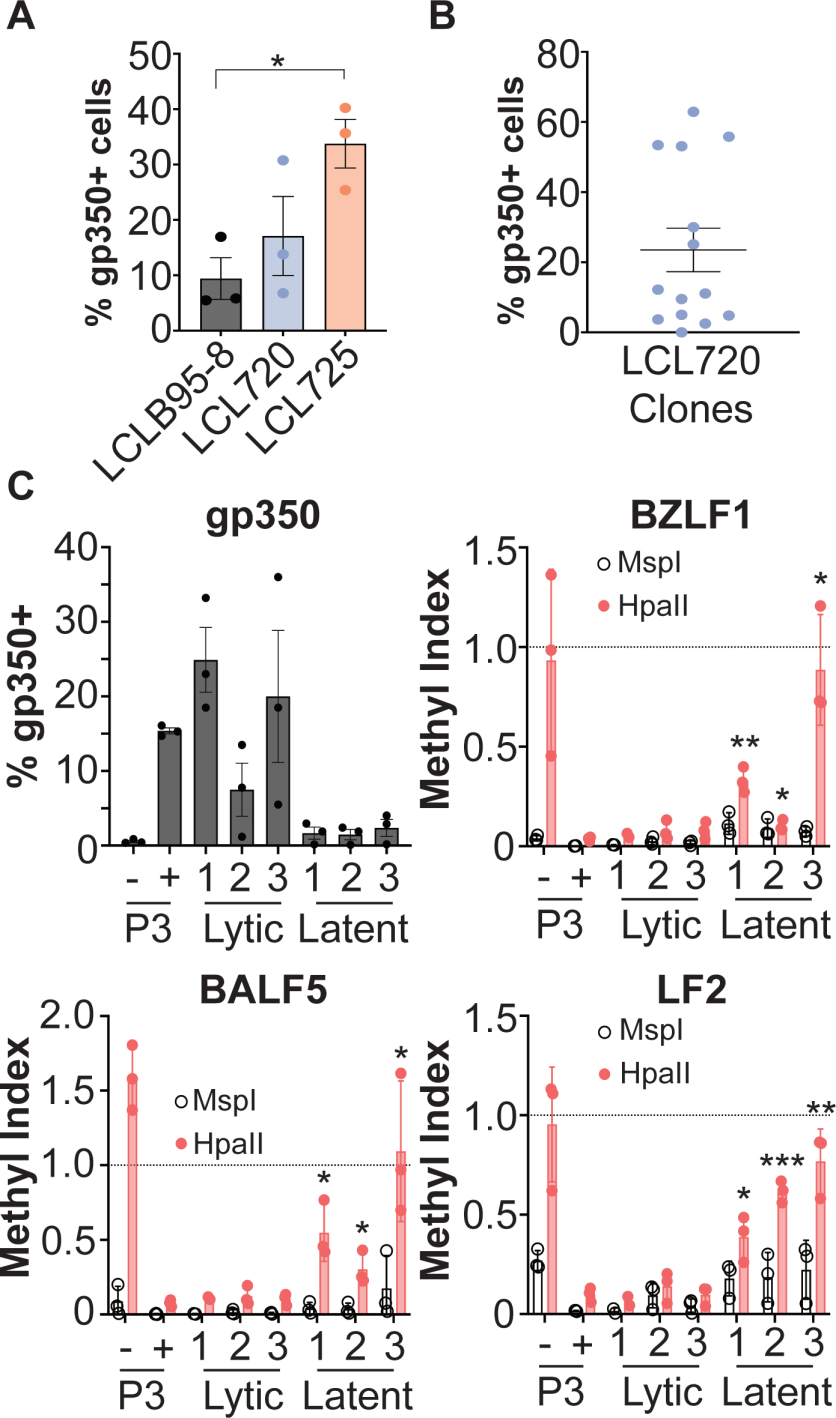
LCLs derived from strains 720 and 725 are spontaneously lytic. (**A**) Gp350 FACS analysis of bulk, donor-matched LCLs derived from infection with strains B95-8, 720, and 725. There is a significant increase in gp350-positive cells in LCL725 compared to LCLB95-8 (*P = 0.0136). (**B**) Gp350 FACS analysis of LCL720 clones derived from infection with a limiting dilution of strain 720. (**C**) gp350 FACS (top left) paired to methyl-qPCR of CpG sites within the viral *BZLF1*, *BALF5*, and *LF2* promoters of three lytic and three latent LCL720 clones. A methylation index of 1 indicates methylation, an index of 0 indicates no methylation. MspI digests are an internal control as this enzyme digests at the CCGG CpG site regardless of methylation status, so it should always be near 0. In contrast, HpaII only digests when the site is unmethylated. P3 (−) = untreated P3HR1 cells, P3 (+) = P3 HR1 cells treated with 50 nM 4HT for 24 h. *=*P* < 0.05, **=*P* < 0.005, ***=*P* < 0.0005 in comparison to P3HR1 + 4HT by unpaired *t* test.

After we established LCLs derived from infection with the new BL-derived EBV strains, we tested if the spontaneous lytic phenotypes observed in the BL background were also apparent in the LCL background. Indeed, we detected gp350 expression by FACS in the 720 and 725 LCLs (Fig. 6A). Thus, the spontaneous lytic phenotype present in the BL cell background transfers to the LCL background upon infection, which suggests that the spontaneous lytic phenotype is encoded by the EBV strains.

Interestingly, when measuring gp350 expression in donor-matched bulk LCLs (i.e., PBMCs from the same human donor were infected with the different strains and transformed into LCLs), we observed a wide variation in gp350 expression in LCL720 (Fig. 6A). To investigate this phenotype, we infected PBMCs with a limiting dilution of strain 720 (i.e., the minimum volume of virus that transformed PBMCs into LCLs), which resulted in clonal LCLs derived from the same blood donor. When we analyzed gp350 expression in these LCL720 clones, we observed a mixture of lytic (gp350+) and latent (gp350−) clones (Fig. 6B). These results explain the wide distribution of gp350 expression in the bulk 720 LCLs observed in Fig. 6A.

We then employed the methyl-qPCR assay to determine if the differential promoter methylation present in lytic vs latent BL720 is also present in these lytic vs latent LCL720 clones. We found that in general, the lytic LCL720 clones have a lower methylation index than the latent LCL720 clones (Fig. 6C). However, though statistically significant, latent clone 2 does not have as strong of a methylation phenotype as the other two latent clones, especially at the *BZLF1* CpG site. Thus, it appears that differential lytic promoter methylation also affects the phenotypic outcome of LCLs infected with strain 720. However, the mechanism governing when a promoter becomes methylated vs remains unmethylated upon LCL generation is unclear. Taken together, these data indicate that the spontaneous T1 lytic phenotype present in BL720 transfers to LCLs generated by infection with strain 720, suggesting that the spontaneous lytic phenotype is encoded by the virus. The diverging phenotypes upon limiting dilution infection of PBMCs from the same human donor indicate that there may be host regulation of the phenotype as well.

### Viral genetic analyses reveal candidates for T1 lytic driver genes

**Fig 7:**
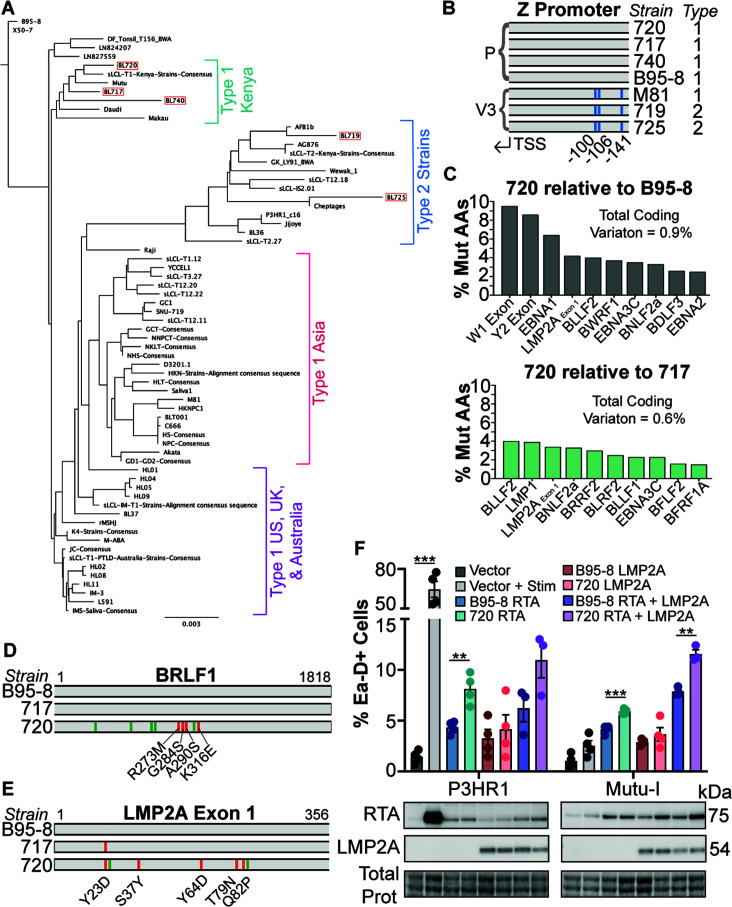
Strain 720 viral genetic analyses. (**A**) Phylogenetic tree of publicly available EBV genomes and the new BL strains from this study (boxed in red) rooted to B95-8. Because some published sequences are very closely related, if the viral genetic sequences were more than 90% similar, they were aligned into a consensus sequence and this consensus was used in the final alignment to build the tree (these instances are notated with “consensus” in the tree). Similar groups are annotated by EBV type and geographic origin. (**B**) Z promoter variant analysis of the new BL-derived strains compared to published strains. (**C**) The top 10 most polymorphic coding regions between strain 720 compared to B95-8 (top) and strain 720 compared to strain 717 (bottom). The percentage of mutated amino acids was calculated based on the available coding sequence length. Total coding variation (mutated amino acids as a percentage of the available coding genome) is noted. (**D**) Coding (red) and non-coding (green) SNPs in strain 720 *BRLF1* compared to strains 717 and B95-8. The coding SNPs are annotated. (**E**) Similar to panel (D) but with the *LMP2A* exon 1 sequence. The Y23D SNP is present in both strains 717 and 720 compared to B95-8. (**F**) Top- FACS analysis of Ea-D induction 1 day after overexpression of the indicated viral proteins. For vector, RTA, and LMP2A overexpression, 4 µg of the construct was transfected. For RTA + LMP2A overexpression, 2 µg of each construct was transfected. “Stim” for P3HR1 was 50 nM 4-HT and 10 µg/ mL anti-IgM for Mutu-I. **=*P* < 0.005, ***=*P* < 0.0005. Bottom- representative western blot from the samples above.

Since the data presented thus far indicate that the T1 spontaneous lytic phenotype is likely encoded by strain 720, we investigated the genome of strain 720 and the other new strains described in this study compared to published EBV genomes. A high-level phylogenetic analysis shows that strains 717, 720, and 740 cluster with other T1 strains from Kenya. Strains 719 and 725 form a cluster with other T2 strains regardless of geographical origin (Fig. 7A). Although the main genetic difference between T1 and T2 strains are the EBNA2 and EBNA3 genes (37), the Zp-V3 polymorphism is also found in all T2 and some rare T1 strains like M81 (3, 56). Zp-V3 is known to enhance lytic activity in strains that contain it (3), so we investigated if strain 720 might be a rare T1 Zp-V3 strain. We found that neither strain 720 nor any of the other new T1 strains contain the Zp-V3 polymorphism (Fig. 7B). This indicates that there is an undescribed viral genetic determinant that is not dependent on Zp-V3 that leads to this T1 lytic phenotype.

We next made direct comparisons of the strain 720 coding genome to the standard lab strain B95-8 and to the close genetic relative, but latent, strain 717. These comparisons revealed that, overall, there are more coding mutations between strains 720 and B95-8 (coding SNPs in 0.9% of total available coding sequence) compared to 720 and 717 (coding SNPs in 0.6% of total available coding sequence) (Fig. 7C), which is expected given that 720 and B95-8 are more genetically divergent (Fig. 7A). However, some coding regions were highly polymorphic in both comparisons, such as LMP2A exon 1, BLLF2, and EBNA3C (Fig. 7C). Although BZLF1 and BRLF1 are not among the most polymorphic coding regions in these comparisons, we also investigated coding mutations present in these genes given their importance in the induction of the EBV lytic cycle. Strains 720, 717, and B95-8 have identical BZLF1 coding sequences (Fig. S4, the SNP in B95-8 is non-coding). However, there are four coding mutations in strain 720 BRLF1 compared to B95-8 and 717: R273M, G284S, A290S, and K316E (Fig. 7D). Although LMP2A is typically considered a latency gene, we were intrigued that exon 1 of the gene is one of the most polymorphic coding regions (Fig. 7C) given that BL720 is in a latency III state (Fig. 2C) and that LMP2A has been described to enhance lytic activity (3). There are five coding mutations in 720 LMP2A exon 1 compared to B95-8 and exon 4 compared to 717 (Fig. 7E). Of these coding SNPs, we noted that serine 37 of B95-8/717 is mutated to tyrosine in 720.

We next investigated if RTA or LMP2A from strain 720 is sufficient to increase lytic activity relative to those proteins from the B95-8 background. To test this hypothesis, we generated the four 720 RTA coding mutations in a plasmid encoding B95-8 *BRLF1* and synthesized plasmids containing either B95-8 LMP2A or 720 LMP2A. We overexpressed these constructs in P3-ZHT and Mutu-I cells. We chose to include P3-ZHT cells because they are highly inducible in the lytic cycle. However, because P3HR1 is a T2 Zp-V3 strain, we also included Mutu-I which is a tightly latent BL line that contains a T1 Zp-P EBV strain and is closely related to BL720 (Fig. 7A). Overexpression of both B95-8 RTA and RTA with the 720 coding mutations induces expression of the endogenous lytic Ea-D protein in P3-ZHT and Mutu-I cells, but the 720 background does so slightly, albeit significantly, more (Fig. 7F; Fig. S5 for data derivation). There was no significant difference in lytic induction by LMP2A from either strain in either cellular background. However, when RTA and LMP2A from the same strains were co-expressed, we observed higher lytic induction overall and significantly more lytic cells induced by 720 RTA + LMP2A compared to B95-8 RTA + LMP2A. These results suggest that 720 RTA polymorphisms increase lytic activity and that this activity is enhanced when the virus is in a latency III state where LMP2A is expressed.

### Cellular factors associated with the T1 lytic phenotype

**Fig 8:**
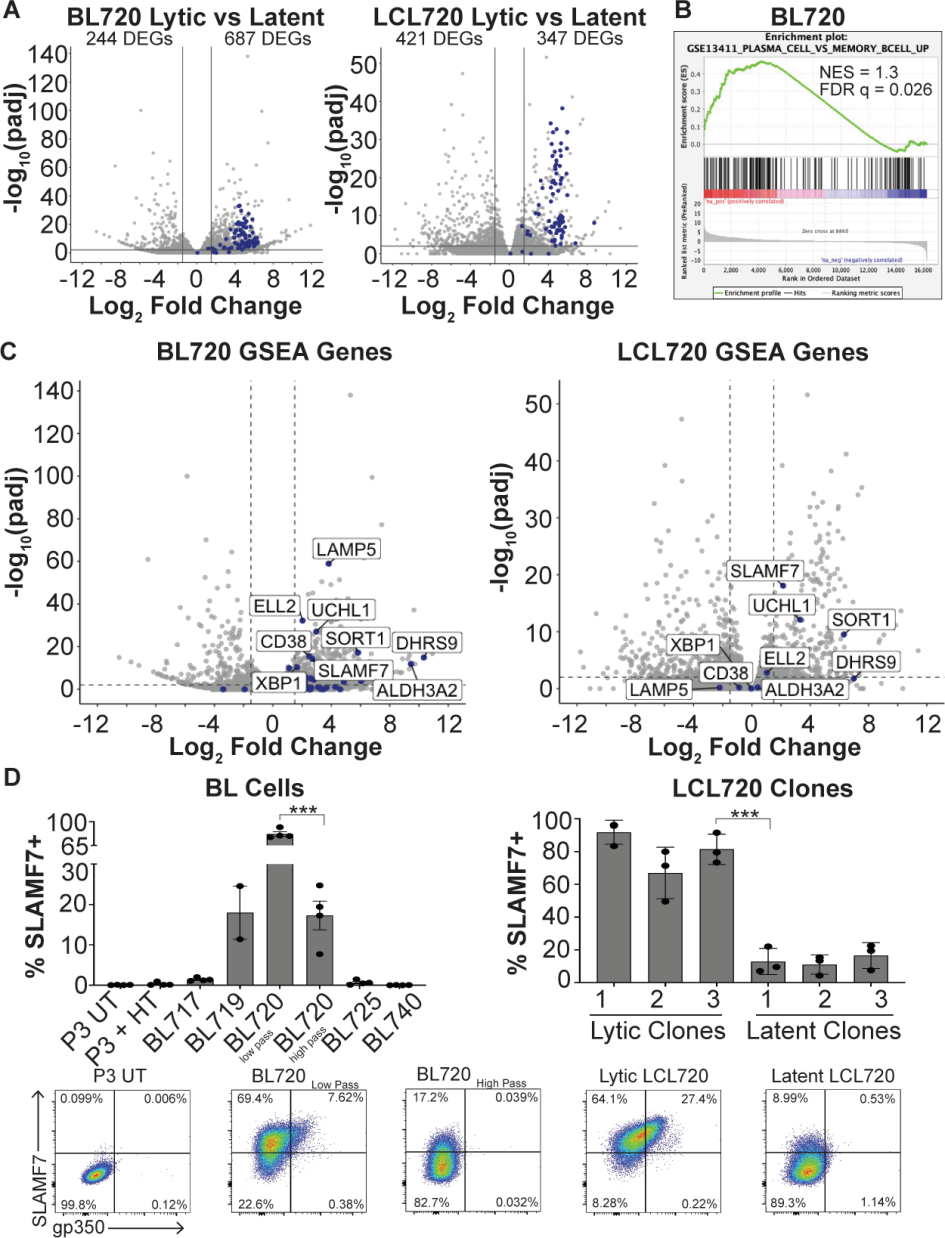
Cellular markers of T1 spontaneous lytic phenotype. (**A**) Volcano plots of differentially expressed genes observed from RNA sequencing of low passage, lytic BL720 (*n* = 3) compared to high passage, latent BL720 (*n* = 3) and lytic LCL720 clones (*n* = 3), and latent LCL720 clones (*n* = 3). For both of these analyses, the human genetic background is the same, so these DEGs are the result of the lytic vs latent viral phenotypes. Host DEGs are in gray, EBV DEGs are in blue. The number of genes downregulated in lytic (left side of plots) and upregulated in lytic (right side of plots) are notated. Cutoffs for DEGs are 1.5 log_2_FoldChange and *P*-adjusted <0.01. (**B**) GSEA analysis of BL720 RNA sequencing data revealed that GSE13411 (plasma cell vs memory B cell up) is significantly enriched in lytic BL720 compared to latent BL720. (**C**) Left- Genes from GSE13411 in B plotted on the BL720 volcano. Genes from the set are in blue and the genes that have >1.5 log_2_FoldChange and p-adjusted <0.01 are labeled. Right- the most significantly enriched genes from BL720 are plotted on the LCL720 volcano. Of these genes, *SLAMF7*, *UCHL1*, and *SORT1* are significantly enriched in lytic BL720 at the >1.5 log_2_FoldChange and *P*-adjusted <0.01 cutoffs. (**D**) SLAMF7 (CD319) and gp350 FACS analysis in BL cells (left) and LCL720 clones (right). There is a significant reduction in SLAMF7 surface expression in high passage, latent BL720 compared to low passage, lytic BL720. Likewise, there is a significant decrease in expression in the latent LCL720 clones compared to the lytic LCL720 clones (***P < 0.0006). Representative FACS plots from these analyses are displayed at the bottom.

Thus far, we have established that the T1 spontaneous lytic phenotype found in BL720 is maintained at a homeostatic level in culture (Fig. 4) and can be transferred to LCLs infected with strain 720 (Fig. 6), but also that the lytic phenotype silences over time (Fig. 2). These results suggest that the phenotype is driven both by viral and host factors. To investigate the host factors that are involved in the regulation of the phenotype, we performed RNA sequencing on low passage lytic BL720 compared to high passage latent BL720 as well as the lytic vs latent LCL720 clones described in Fig. 6B. Differential gene expression analysis revealed a surprising number of differentially expressed genes (Tables S1 and S2), especially considering that the samples within each group are from the same human genetic backgrounds, meaning that the differential gene expression is driven by the lytic vs latent state of the virus. In the BL720 analysis, there are 244 genes downregulated and 687 genes upregulated in lytic compared to latent BL720. In the LCL720 analysis, there are 421 genes downregulated and 347 genes upregulated in the lytic compared to the LCL720 clones. Additionally, all the EBV genes that were captured by sequencing are upregulated in the lytic groups for both analyses (Fig. 8A, EBV genes in blue).

Because of the high number of DEGs, we performed gene set enrichment analysis (GSEA) to guide our interpretation of the results (Table S3). One of the significantly enriched gene sets for BL720 lytic vs latent includes genes upregulated in plasma cells vs memory B cells (GSE13411, Fig. 8B). However, this gene set is not significantly enriched in the LCL720 lytic vs latent analysis (Fig. S6). This result was intriguing because EBV-infected BL cells are typically thought to be memory B cell-like and plasma cell differentiation is a known trigger for lytic reactivation (11, 14–16, 57, 58). Some prototypical plasma cell genes were significantly enriched in the lytic BL cells (i.e. *XBP1* and *CD38*), but the gene expression pattern of the lytic subset was not that of bona fide plasma cells. This suggests that the lytic cells have some plasma cell characteristics that enhance lytic reactivation but are not necessarily true plasma cells. We further investigated this gene set by plotting the genes from the set on the BL720 lytic vs latent RNA seq results and identifying the top significantly enriched genes (Fig. 8C, left, genes from gene set in blue). We then cross-referenced these hits with the LCL720 DEGs and found that three of them (*SLAMF7*, *UCHL1*, and *SORT1*) are significantly enriched in lytic LCL720 (Fig. 8C, right). Of these three conserved genes upregulated in the lytic cells between both data sets, *SLAMF7* encodes for a surface-expressed protein SLAMF7/CD319, which is a robust marker of plasma cells, especially in multiple myeloma and plasmablastic lymphoma (59–61). We therefore decided to validate this hit through FACS analysis for SLAMF7.

We found that SLAMF7 is indeed a dependable marker of spontaneous lytic EBV replication (Fig. 8D). SLAMF7 is highly expressed on low passage lytic BL720 cells and this expression significantly decreases in high passage latent BL720 cells. BL719 also displays high SLAMF7 expression; however, BL725 does not. Additionally, nearly all the gp350(+) cells in BL719 and BL720 are also SLAMF7(+). Importantly, P3-ZHT cells induced into the lytic cycle with 4-HT treatment do not express SLAMF7, indicating that this marker is specific to spontaneously lytic cells. This result was also validated in the LCL720 clones (Fig. 8D, right). The lytic LCL720 clones express significantly more SLAMF7 compared to the latent clones and the gp350(+) cells within the LCL populations are all SLAMF7(+). It is notable that the percentage of SLAMF7 expressing cells in both the BL lines and LCL720 clones is higher than the percentage of gp350 expressing cells, indicating that there are gp350(−) cells that also express SLAMF7. Taken together, these results indicate that within a heterogeneous bulk population of EBV-infected cells, SLAMF7 is a robust and reliable marker not only of lytic cells but also of cells that are primed to spontaneously enter the lytic cycle.

## DISCUSSION

The discovery of the spontaneous lytic T1 Zp-P strain 720 is an important contribution to the EBV research field as it alters the paradigm of what we consider to be prototypical EBV phenotypes. Our characterizations of the lytic phenotypes in strains 719, 720, and 725 add to the growing body of evidence that specific EBV genotypes can result in spontaneous entry into the lytic cycle (3, 4, 24, 38, 62). Additionally, our characterization of the lytic and latency patterns in these BL lines increases support to the findings that BL does not exist exclusively in a latency I state (46–49, 63). We have also determined that these altered latency states enhance spontaneous lytic replication through LMP2A cooperation with RTA. Because it can signal through the B-cell receptor signaling pathway, LMP2A has been reported to contribute to lytic replication under certain circumstances (3). However, the mechanism by which LMP2A enhances RTA activity in the strain 720 background is unclear and complicated by host cell factors that also appear critical to the resolution of the phenotype.

It is notable that the spontaneous lytic phenotype described in strains 719, 720, and 725 produces much fewer viral particles compared to induced lytic systems (Fig. 1). Additionally, this low, tonic level of viral production does not appear to kill the productive cells, as indicated by the sorting and proliferation tracking experiments in Fig. 4. This phenotype appears analogous to the “smoldering lytic” phenotype that has been described in another herpesvirus, human cytomegalovirus (CMV) (64). Persistent viral shedding and cell survival have also been described in human coronavirus 299E infections where the transcription factor ZBTB7A controls oxidative stress to enable this infection outcome (65). Thus, the spontaneous lytic EBV phenotypes described in this study may be representative of an underappreciated form of low-level replication that occurs across viral species.

Another surprising discovery made in this study is that lytic replication is correlated with lower c-Myc stability, even in the context of the immunoglobulin-*Myc* translocations characteristic of BL. Our finding that lytic BL720 has significantly higher c-Myc RNA expression compared to latent BL720 (Log_2_FC = 0.695, padj = 0.007) even though c-Myc protein expression is diminished in the lytic states adds further support that c-Myc protein is turned over more quickly through the proteasomal degradation pathway in cells undergoing lytic replication. This finding is complementary to the recently described results that c-Myc helps control the induction of the EBV lytic cycle (20). However, it is unclear whether low c-Myc protein levels help trigger lytic reactivation or whether lytic replication leads to c-Myc degradation and how these low steady-state c-Myc levels impact cell survival and tumor progression.

We were also intrigued that we were able to generate LCLs more efficiently through infection with strain 725 compared to B95-8 even though 725 is a T2 strain which are known to be less transforming *in vitro* (55). However, we noted that similar to previous reports about T2 LCLs (23), the resulting 725 LCLs were less robust than a typical T1 LCL; they did not grow as quickly and were more prone to die out in culture. Even so, the ability to efficiently generate an LCL from a T2 strain is notable since there does not appear to be a defect in T2 strains to infect and establish latency or cause disease *in vivo* (66, 67). The stark difference in transformation ability between the T2 strains 719 and 725 is also of interest. Detection of Wp transcripts 3 days after infection with strain 719 indicates that it infects cells efficiently, but the lack of proliferating cells 7 days after infection suggests an early block in transformation compared to strain 725. A more thorough examination of LCLs derived from strain 725 and comparisons of strains 719 and 725 early after infection may help elucidate how T2 strains are able to establish latency *in vivo* despite the alterations in the EBNA2/3 genes that make them less transforming *in vitro*.

We have provided a thorough overview of the behaviors of strain 720 *in vitro*; however, many questions remain about how spontaneous lytic EBV strains affect the host and the development of EBV-associated diseases. Considering that this T1 lytic phenotype was first observed in early passage BL tumor cell isolates, it is reasonable to hypothesize that the phenotype may also occur *in vivo*, though perhaps at a lower level when there is an intact immune response. Our collaborators developed NSG-BL-Avatar mouse models using the BL lines described in this study and found that BL720 Avatars developed larger tumors, had lower survival, and also had a higher proportion of ZTA+ cells compared to the other BL strains (25), suggesting that the T1 Zp-P spontaneous lytic phenotype can occur *in vivo* and that it may affect disease outcome. It is also intriguing that BL720 was derived from an endemic BL patient that did not survive (25). However, larger epidemiological screening studies of patients with endemic BL and other EBV-associated diseases will be required to confidently determine whether spontaneous lytic EBV strains are more common than we currently appreciate and if they affect patient outcomes.

The clear correlation between SLAMF7/CD319 expression and spontaneous lytic activity that we described in this study may offer an option for such high throughput epidemiological studies. SLAMF7 expression is currently used as a marker for multiple myeloma and plasmablastic lymphomas (59, 60). In the context of EBV-associated diseases, SLAMF7 could be a particularly useful maker when screening for cells that have the potential to spontaneously enter the lytic cycle since the proportion of cells actively displaying EBV lytic antigens, such as gp350, in a patient biopsy with a limited number of cells may be relatively low. Additionally, Elotuzumab is an anti-SLAMF7 monoclonal antibody that is FDA-approved for the treatment of multiple myeloma (68, 69), raising the possibility that such drugs could also be used in the treatment of EBV-associated tumors with high SLAMF7 expression.

This study has provided a detailed description of viral and host factors that lead to a novel Type 1 (T1) Zp-P EBV spontaneous lytic phenotype. The resolution of this phenotype is multifactorial and dependent on viral polymorphisms as well as host cell state. It is therefore unsurprising that a T1 Zp-P spontaneous lytic phenotype has not been described until now. The ability to culture patient tumors then characterize them with the molecular methods described in this study has increased our appreciation for EBV strain diversity and heterogeneous disease states. Continued generation of BL and other EBV+ cell lines may enable the discovery of additional lytic T1 Zp-P EBV strains, lead to a better understanding of the factors that lead to T1 lytic phenotypes, and help determine how spontaneous lytic EBV replication contributes to EBV-associated diseases.

## Data Availability

The RNA-Seq data have been uploaded to the Database of Genotypes and Phenotypes (dbGaP) under accession number phs001282.v4.p1. The newly described EBV genome sequences have been uploaded to NCBI GenBank with the following accession numbers: strain 717, OR652419; strain 719, OR652420; strain 720, OR652421; strain 725, OR652422; strain 740, OR652423.

## SUPPLEMENTAL MATERIAL

Supplemental figures and legends mbio.02204-23-s0001.pdf Figures S1 to S6 and legends for all supplemental material.

Table S1 mbio.02204-23-s0002.xlsx Lytic versus latent BL 720 differential gene expression.

Table S2 mbio.02204-23-s0003.xlsx Lytic versus latent LCL 720 differential gene expression.

Table S3 mbio.02204-23-s0004.xlsx GSEA of BL and LCL 720 differential gene expression.

Video S1 mbio.02204-23-s0005.mp4 Live cell video of BL720 sorted gp350-positive cells.

Video S2 mbio.02204-23-s0006.mp4 Live cell video of BL720 sorted gp350-positive cells.

Video S3 mbio.02204-23-s0007.mp4 Live cell video of BL720 sorted gp350-positive cells.

Video S4 mbio.02204-23-s0008.mp4 Live cell video of BL720 sorted gp350-positive cells.

Video S5 mbio.02204-23-s0009.mp4 Live cell video of BL720 sorted gp350-positive cells.

Video S6 mbio.02204-23-s0010.mp4 Live cell video of BL720 sorted gp350-negative cells.

ASM does not own the copyrights to Supplemental Material that may be linked to, or accessed through, an article. The authors have granted ASM a non-exclusive, world-wide license to publish the Supplemental Material files. Please contact the corresponding author directly for reuse.
