# Supplementary material for: Viral and host factors drive a type 1 Epstein–Barr virus spontaneous lytic phenotype
Source: mBio. 2023 Nov 16;14(6):e02204-23. doi: 10.1128/mbio.02204-23 (PMC10746244; doi:10.1128/mbio.02204-23)
Supplement: Supplemental figures and legends — Figures S1 to S6 and legends for all supplemental material. [file mbio.02204-23-s0001.pdf]

Supplemental Figure 1

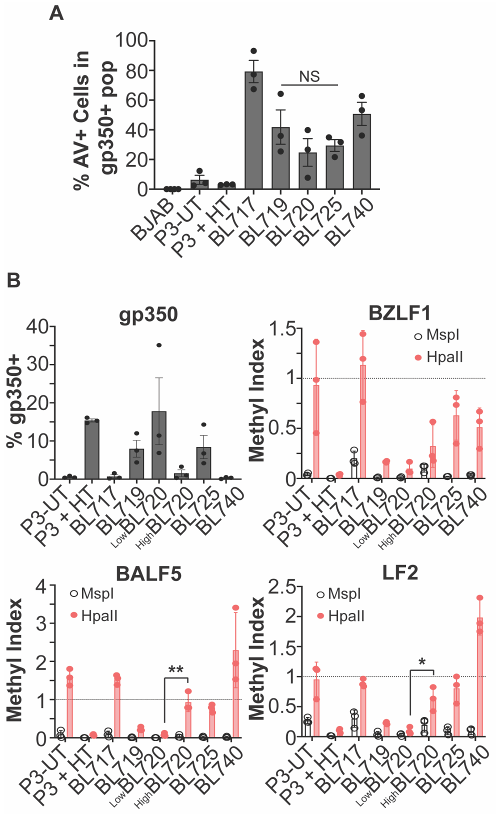

A) Percentage of gp350+ cells that are also Annexin V positive as measured by FACS. Note that the high percentage of double positive cells for BL717 and BL740 is due to the low number of gp350(+) cells in the bulk population. Additionally, it appears that the gp350(+) cells in BL717 and BL740 are mostly Annexin V(+), suggesting that they tend to die resulting in an overall latent phenotype for these lines. NS = no significance difference by unpaired t-test.

B) gp350 FACS (top left) paired to methyl-qPCR of CpG sites within the viral *BZLF1*, *BALF5*, and *LF2* promoters. A methylation index of 1 indicates methylation, an index of 0 indicates no methylation. MspI digests are an internal control as this enzyme digests at the CCGG CpG site regardless of methylation status, so it should always be near 0. In contrast, HpaII only digests when the site is unmethylated. Low passage BL720 is < p13, high passage BL720 is > p158. * = p < 0.05, ** = p < 0.005 by unpaired t-test.

Supplemental Figure 2

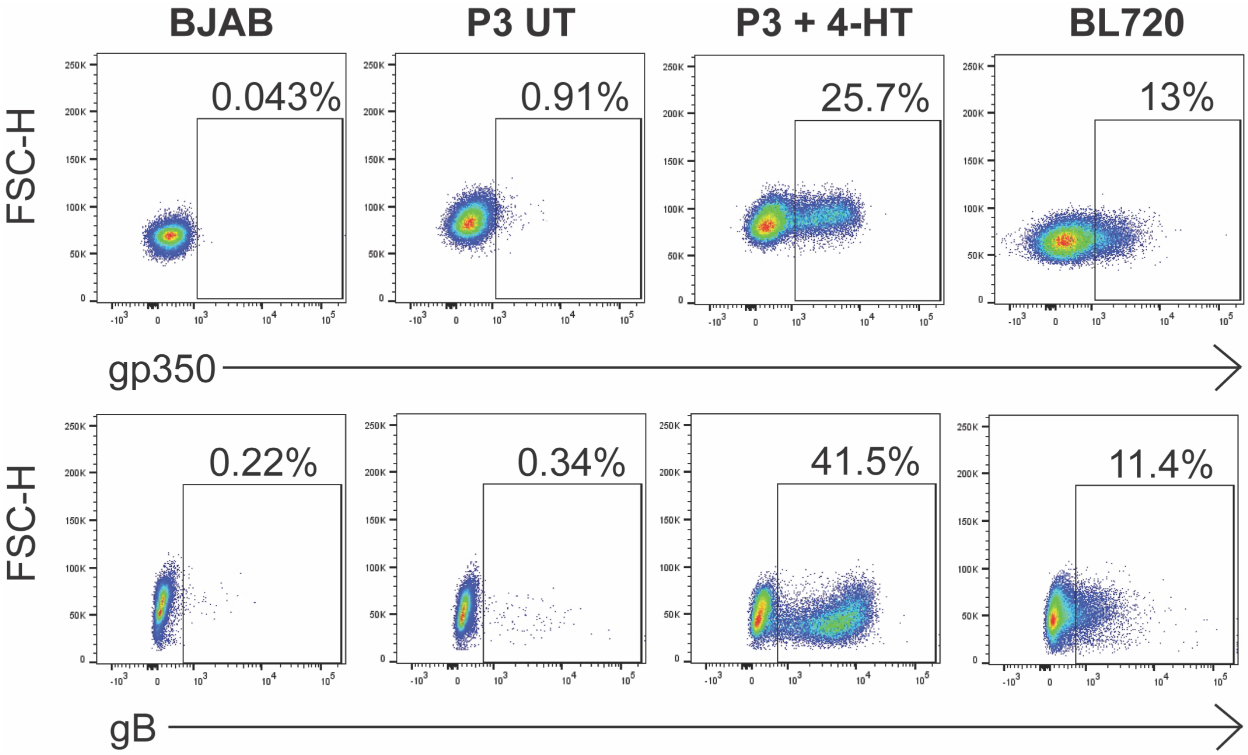

FACS analysis of gp350 and gB/ gp110 expression in BJAB, UT P3HR1, 4-HT stimulated P3HR1, and BL720 cells. Surface gp350 was detected as previously described. Because it is primarily expressed on the ER and perinuclear membranes, gB/ gp110 was detected through intracellular staining with 1 μg of 5B2 antibody followed by 1 μg of highly-cross adsorbed anti-mouse Alexa 488. Gates were set using an isotype control.

Supplemental Figure 3

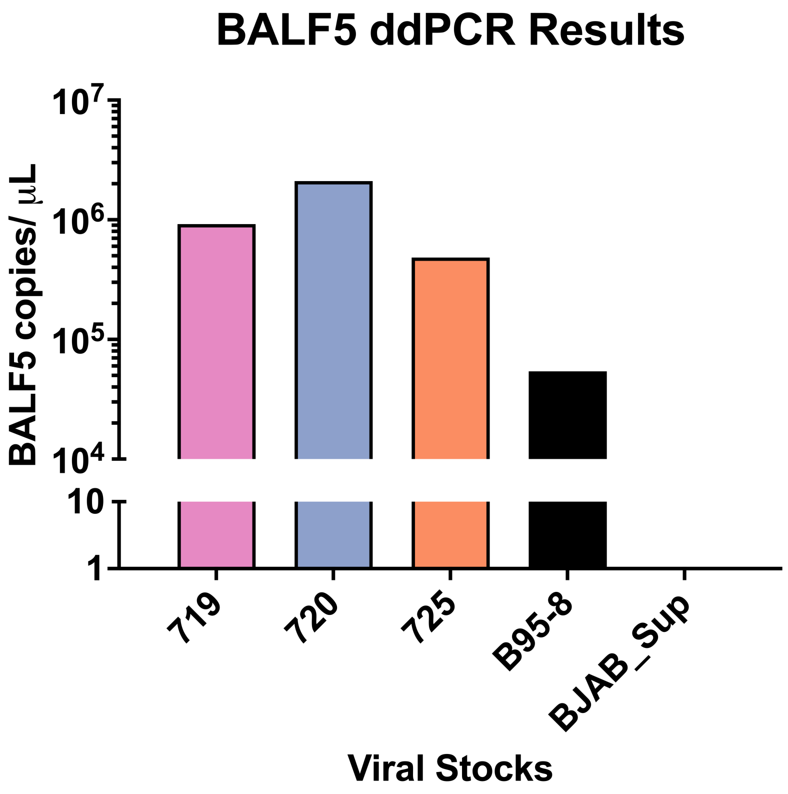

Digital droplet PCR quantification of BALF5 copies/ μL of the indicated viral stocks and BJAB cell supernatant (EBV negative control).

Supplemental Figure 4

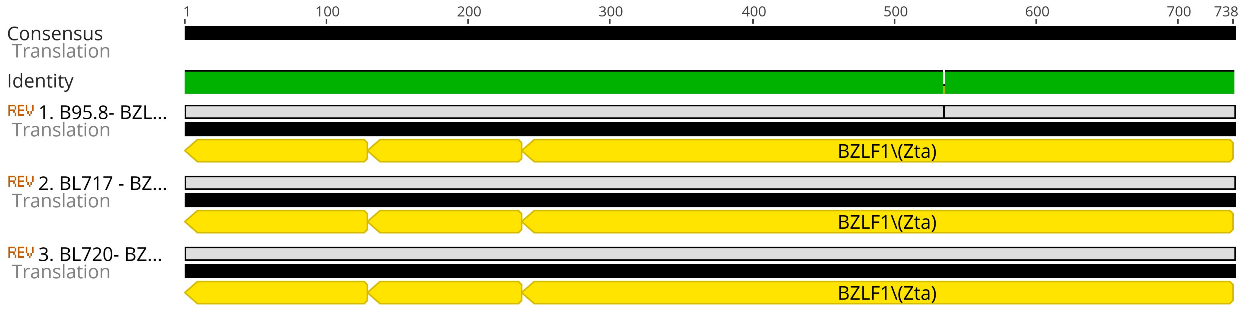

Alignment of the B95-8, 717, and 720 BZLF1 coding regions shows that the protein sequences are identical. The SNP present in B95-8 is noncoding.

Supplemental Figure 5

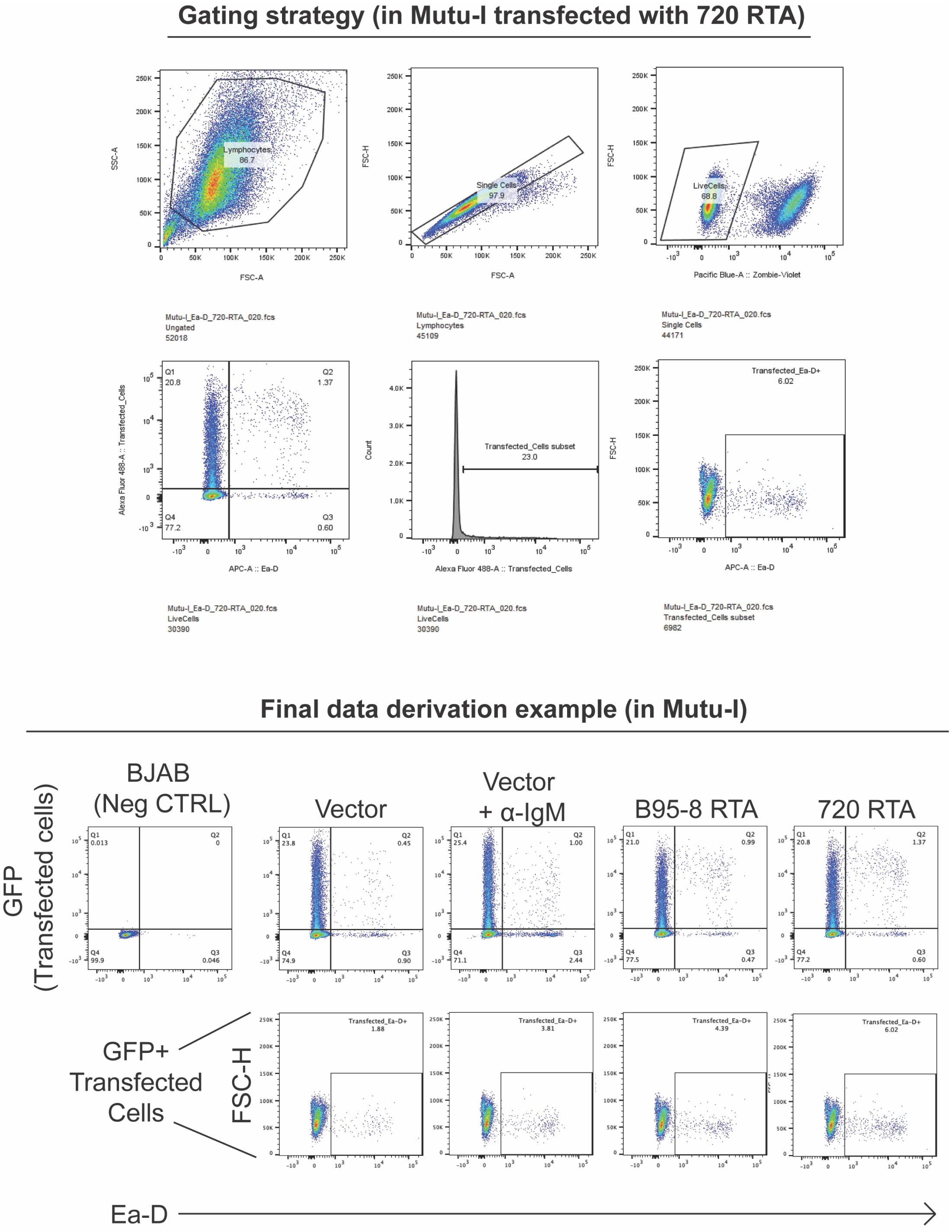

Example of FACS gating strategy and final data derivation for RTA and LMP2A overexpression experiments in Figure 7F.

Supplemental Figure 6

Table: GSEA Results Summary

Dataset	Lytic-LCL_vs_Latent-LCL_GSEA.rnk_collapsed
Phenotype	NoPhenotypeAvailable
Upregulated in class	na_neg
GeneSet	GSE13411_PLASMA_CELL_VS_MEMORY_BCELL_UP
Enrichment Score (ES)	-0.2897784
Normalized Enrichment Score (NES)	-0.88532555
Nominal p-value	0.75944585
FDR q-value	0.75944585
FWER p-Value	0.603

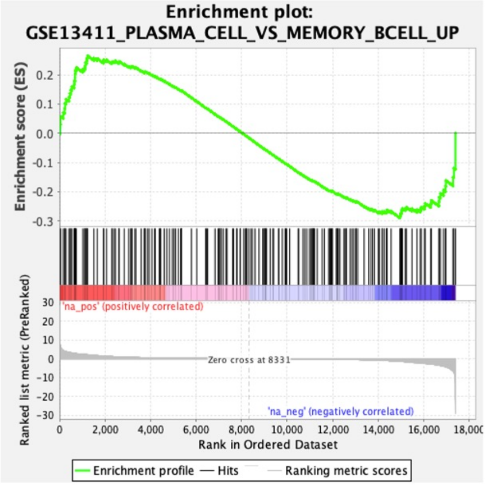

Fig 1: Enrichment plot: GSE13411_PLASMA_CELL_VS_MEMORY_BCELL_UP
Profile of the Running ES Score & Positions of GeneSet Members on the Rank Ordered List

GSEA GSE13411 enrichment plot for Lytic LCL720 vs Latent LCL720 reveals that this gene set is not differentially expressed in this comparison.

Supplemental Video 1

Live cell microscopy video #1 of gp350(+) BL720 cells. Images were captured every 30 minutes for 5 days after gp350 sorting.

Supplemental Video 2

Live cell microscopy video #2 of gp350(+) BL720 cells. Images were captured every 30 minutes for 5 days after gp350 sorting.

Supplemental Video 3

Live cell microscopy video #3 of gp350(+) BL720 cells. Images were captured every 30 minutes for 5 days after gp350 sorting.

Supplemental Video 4

Live cell microscopy video #4 of gp350(+) BL720 cells. Images were captured every 30 minutes for 5 days after gp350 sorting.

Supplemental Video 5

Live cell microscopy video #5 of gp350(+) BL720 cells. Images were captured every 30 minutes for 5 days after gp350 sorting.

Supplemental Video 6

Live cell microscopy video of gp350(-) BL720 cells. Images were captured every 30 minutes for 5 days after gp350 sorting.

Supplemental Table 1

Excel workbook of differentially expressed genes in lytic BL720 compared to latent BL720 from RNA sequencing experiment.

Supplemental Table 2

Excel workbook of differentially expressed genes in lytic LCL720 clones compared to latent LCL720 clones from RNA sequencing experiment.

Supplemental Table 3

Excel workbook summary of significant gene sets from BL720 and LCL720 RNA sequencing GSEA analyses.
